# A formulation containing *Cymbopogon flexuosus* essential oil: improvement of biochemical parameters and oxidative stress in diabetic rats

**DOI:** 10.3762/bjnano.16.48

**Published:** 2025-05-07

**Authors:** Ailton Santos Sena-Júnior, Cleverton Nascimento Santana Andrade, Pedro Henrique Macedo Moura, Jocsã Hémany Cândido dos Santos, Cauãn Torres Trancoso, Eloia Emanuelly Dias Silva, Deise Maria Rego Rodrigues Silva, Ênio Pereira Telles, Luiz André Santos Silva, Isabella Lima Dantas Teles, Sara Fernanda Mota de Almeida, Daniel Alves de Souza, Jileno Ferreira Santos, Felipe José Aidar Martins, Ana Mara de Oliveira e Silva, Sandra Lauton-Santos, Guilherme Rodolfo Souza de Araujo, Cristiane Bani Correa, Rogéria De Souza Nunes, Lysandro Pinto Borges, Ana Amélia Moreira Lira

**Affiliations:** 1 Department of Pharmaceutical Sciences, Federal University of Sergipe, São Cristóvão 49100-000, Sergipe, Brazil; 2 Department of Biology, Federal University of Sergipe, São Cristóvão 49100-000, Sergipe, Brazil; 3 Department of Physiology, Federal University of Sergipe, São Cristóvão 49100-000, Sergipe, Brazil; 4 Department of Morphology, Federal University of Sergipe, São Cristóvão 49100-000, Sergipe, Brazil; 5 Department of Physical Education, Federal University of Sergipe, São Cristóvão 49100-000, Sergipe, Brazil; 6 Department of Nutrition, Federal University of Sergipe, São Cristóvão 49100-000, Sergipe, Brazil

**Keywords:** diabetes mellitus, essential oil, lemongrass, microemulsion

## Abstract

Diabetes mellitus (DM) is a highly prevalent public health problem characterized by hyperglycemia that causes complications due to the generation of reactive oxygen species and oxidative damage. Studies have shown that essential oils containing citral, such as lemongrass, have various biological activities, including bactericidal, antiviral, antifungal, antioxidant, and hypoglycemic effects. Therefore, this study aims to obtain a microemulsified formulation containing the essential oil of *Cymbopogon flexuosus* (EOCF) and to evaluate its antioxidant and antidiabetic activity in diabetic rats. The microemulsion (ME) was obtained after consulting the corresponding pseudoternary phase diagram and showed stability, isotropy, Newtonian behavior, nanometric size (15.2 nm), and pH 4.2. Both EOCF and the ME showed high antioxidant activity, but the ME resulted in greater antioxidant activity, potentiating the activity of isolated EOCF. Finally, male Wistar rats (3 months old, 200–250 g) with streptozotocin-induced type-1 DM were supplemented with EOCF and ME (32 mg/kg) for 21 days. Both EOCF and ME supplementation resulted in reduced blood glucose levels and improved lipid profiles when compared to the control group. Additionally, the ME was able to provide additional benefits, such as reduced liver damage, improved renal function, reduced systemic inflammation, and increased high-density lipoprotein levels. Overall, the results show that EOCF was efficiently incorporated into the microemulsion, improving its antioxidant activity and showing promising results for use in the treatment of DM via the oral route.

## Introduction

Diabetes mellitus (DM) is one of the main public health problems. It affects around 463 million people worldwide, and it could reach 700 million cases by 2045 [1]. The main causes of DM are related to a deficit in insulin production due to a loss of pancreatic β-cell function (type-1 DM) [1], or due to insulin resistance and a partial deficit in the secretion of this hormone by pancreatic β-cells (type-2 DM).

Various therapies have been used in the treatment of patients with DM including medication using hypoglycemic agents alone or combined with the administration of exogenous insulin, associated with dietary re-education and changes in lifestyle habits, such as physical exercise [2]. Although hypoglycemic agents are widely prescribed in DM therapy, they can result in limited efficacy and cause various adverse reactions such as dyspepsia, abdominal pain, nausea, and lactic acidosis [3], which vary according to the hypoglycemic agent used and the patient’s individual response.

As an alternative to conventional DM treatments, natural sources are being investigated, such as citral, the main compound in the essential oil (EO) extracted from plants of the *Cymbopogon* genus. Both citral and the essential oils of species such as *Cymbopogon flexuosus* (EOCF) and *Cymbopogon citratus* have shown antioxidant, antidiabetic, and antidyslipidemic properties in vivo [4–6]. EOs are volatile components that are sensitive to temperature, light, oxygen, and humidity [7]. Therefore, it is recommended to use them in drug delivery systems, such as nanostructured systems, which are able to guarantee stability and a better therapeutic effect after in vivo administration [8]. Among these systems, microemulsions (MEs) stand out for their ability to increase the solubility, absorption, and bioavailability of lipophilic compounds such as EOs. MEs are transparent, thermodynamically stable, low-viscosity systems containing oil and water stabilized by surfactants, with a very small droplet size (<100 nm), which facilitates their permeation through membranes [9]. In addition, MEs showed increased anti-inflammatory activity, reduced irritation, and improved the stability of EOs in previous studies [10–11].

Thus, from an innovative perspective, the aim of this work is to evaluate the antioxidant and antidiabetic activity of EOCF and a ME containing EOCF. Glycemic and lipid parameters, oxidative damage, and tissue injury in diabetic rats were evaluated.

## Results and Discussion

### Chemical composition of EOCF

The GC-MS analysis results of EOCF are similar to those presented in our previous study [12] and are shown in Table S1 (Supporting Information File 1). In total, 13 compounds were identified, the majority being α-citral (geranial, 53%), β-citral (neral, 19%), and geraniol (12%). EOCF also contained smaller percentages (0.07% to 4.90%) of constituents such as tricyclone, α-pinene, canphene, nonanone, linalool, isogeraniol, isoneral, isocitral, caryophyllene, and geranyl acetate. A study by Adukwu et al. [13] showed that citral (neral and geranial, 89%) and geraniol (8%) were the major constituents of EOCF, followed by linalool (2.7%), corroborating the findings of the present study. However, other minor components were not identified in this study, showing that the composition of the essential oil can change significantly when obtained from different geographical origins.

### Development of microemulsions considering the pseudoternary phase diagram

Various surfactants in different combinations were tested to obtain the ME. In general, non-ionic surfactants are most commonly used because they have a low critical micelle concentration, low toxicity, and greater stability to changes in pH and charge when compared to other classes of surfactants [14]. Another important parameter is the hydrophilic–lipophilic balance (HLB). Surfactants with low HLB values between 3 and 6 generally promote the formation of water/oil (W/O) emulsions, while high HLB values between 8 and 18 predominantly result in O/W emulsions [14]. The HLB values of Eumulgin^®^ CO 40 (14.1) and Tween^®^ 80 (15) are suitable for O/W formulations.

**Figure 1:**
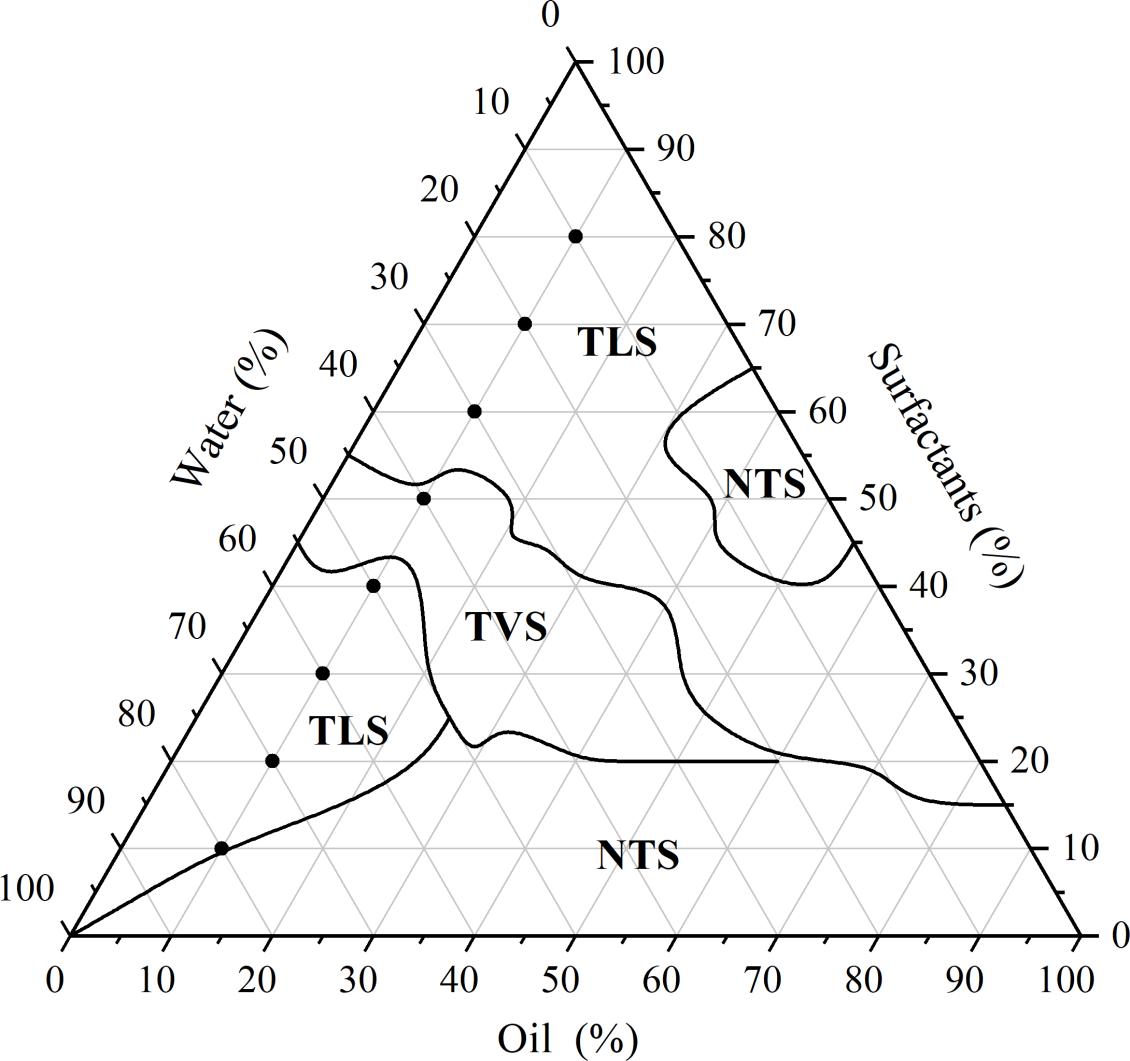
Pseudoternary phase diagram (PTPD) of EOCF, water, and a mixture of the surfactants Eumulgin^®^ CO40/Tween^®^ 80, in a 1:1 ratio. The PTPD shows demarcated regions corresponding to transparent liquid systems (TLS), transparent viscous systems (TVS) and non-transparent systems (NTS). The PTPD also shows formulations obtained with fixed oil phase concentrations (10%).

Among various combinations of the components (Supporting Information File 1, Table S2), the pseudoternary phase diagram (PTPD) that best provided transparent liquid systems was obtained with Eumulgin^®^ CO40/Tween^®^ 80, in a ratio of 1:1 (v/v), containing distilled water as the aqueous phase and EOCF as the oil phase, as shown in Figure 1. Although the PTPD with propylene glycol and Olivem^®^ 300 (1:1, v/v) also showed regions characterized as ME, these regions contained only a low amount of EOCF (1%). Therefore, for the in vivo study, the required amount for oral administration would be high and could be unfeasible. Hence, the pseudoternary phase containing Eumulgin^®^ CO40 and Tween^®^ 80 was selected for the subsequent trials.

In the PTPD in Figure 1, a broad area of transparent systems is observed, suggesting that the use of these components can facilitate the development of nanometer-scale systems such as MEs and liquid crystals [15]. This region was divided into three distinct regions, with a central region of transparent viscous systems (TVS), which may be a region of lyotropic liquid crystals [16–17], and two regions of transparent liquid systems (TLS), which have low viscosity, suggesting the formation of MEs [18]. In general, the PTPD obtained with Eumulgin^®^ CO 40 and Tween^®^ 80 is similar to the PTPDs obtained with other similar surfactants such as shown in [8,19].

Considering the region of formation of transparent liquid systems obtained in the PTPD above, the dilution line containing 10% EOCF was selected, and the spots were prepared individually and monitored for five days (see below Table 1 and Figure 1). After this time, five formulations were classified as TLS suggesting that MEs were obtained. In addition, two SVT and one NTS were also obtained. Among the TLS, point 7 (M7-EOCF) was selected for subsequent tests, as it had the lowest amount of surfactants and the highest proportion of water in its composition.

### Physicochemical characterization of the selected system

Some parameters are fundamental for characterizing nanostructured systems such as MEs, including droplet size, optical properties, and rheological profile. M7-EOCF therefore underwent tests to investigate these parameters. First, the M7-EOCF sample was analyzed by dynamic light scattering, which showed that the system had an average hydrodynamic radius of 15.24 ± 1.27 nm, which is in line with the definition of MEs, having a hydrodynamic radius between 10 and 100 nm [18]. Furthermore, it was observed that the M7-EOCF formulation had a polydispersity index (PDI) of 0.334 ± 0.054, indicating homogeneity (PDI < 0.5) in the droplet size distribution of this system. The literature reports that a PDI below 0.5 also indicates greater physical stability of the obtained formulation [20–21].

The M7-EOCF sample was also examined using polarized light microscopy to investigate its optical properties. Isotropic behavior was observed (Supporting Information File 1, Figure S1) through the visualization of a dark field. In general, microemulsions do not deflect the plane of polarized light since their optical properties are constant in all directions [18].

**Figure 2:**
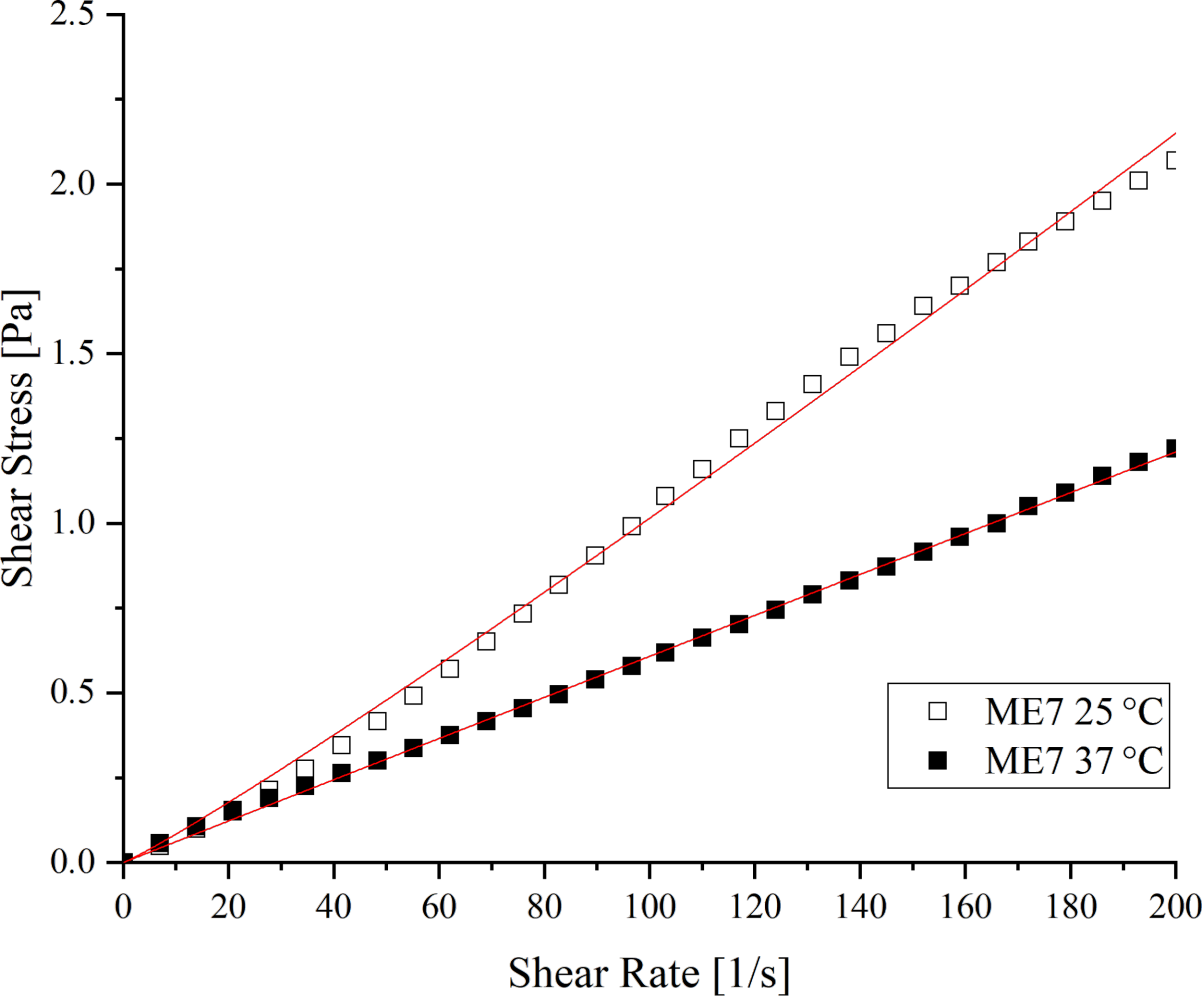
Rheological behavior (shear stress versus shear rate) of the microemulsion M7-EOCF at 25 and 37 °C.

Rheology is the science that studies the flow characteristics and deformation of matter when exposed to different shear forces [22–23]. The parameters of rheological properties are essential in the classification and characterization of semisolids and fluids, and in the greater understanding of drug release from pharmaceutical forms [22]. Thus, the rheological behavior of M7-EOCF at two temperatures (25 and 37 °C) was analyzed. The results were expressed in rheograms (Figure 2) and represent the relationship between the shear stress and the shear rate.

The data were analyzed using the power law (τ = *k*⋅γ*^n^*), where τ is the shear rate, *k* is the consistency index (viscosity), γ is the shear stress, and *n* is the flow index. The flow curves showed a linear correlation (*n* close to 1). For 25 °C, *n* was 1.08, while for 37 °C, it was 0.99 (i.e., Newtonian behavior). *R*^2^ was greater than 0.996 for both tests, indicating that the data fit this model. This is expected for a microemulsion system [18,23–24]. The viscosity at 25 °C was 0.009 ± 0.001 Pa/s. The low viscosity can be justified considering the high water content present in the M7-EOCF formulation [25]. And it can be seen that increasing the temperature caused a decrease in viscosity to 0.006 ± 0.001 Pa/s (at 37 °C).

The low viscosity of MEs is advantageous when taken orally as it facilitates oral application and dose adjustment; it also improves absorption and bioavailability. Additionally, MEs can be administered to humans as self-microemulsifying drug delivery systems (SMEDDSs) delivered in soft gelatin capsules as they are low-viscosity liquids. SMEDDSs are concentrated microemulsions that contain only the oil phase and the surfactant phase (without water). SMEDDSs can form MEs spontaneously through the addition of water and gentle agitation, such as movement of the gastrointestinal tract, and can be formulated into capsules [26–27], improving patient acceptance [28]. The pH value is also capable of inducing changes in stability and chemical phenomena, such as deterioration and oxidation of compounds and bacterial growth, and is a precise and specific measure for quality control of the formulation. The pH found for M7-EOCF was 4.65 ± 0.05. Orally tolerated pH values are between 2 and 10 [29]. In this context, M7-EOCF has a pH value compatible with oral use, thus demonstrating that the formulation will not cause irritation to the mucous membranes.

Altogether, considering its composition (i.e., an oil phase, an aqueous phase, and a surfactant phase), transparent appearance, low viscosity, isotropy, Newtonian behavior, and droplet size on a nanometric scale, it can be said that the M7-EOCF formulation is a microemulsion system [30–31].

### Antioxidant activity

**Figure 3:**
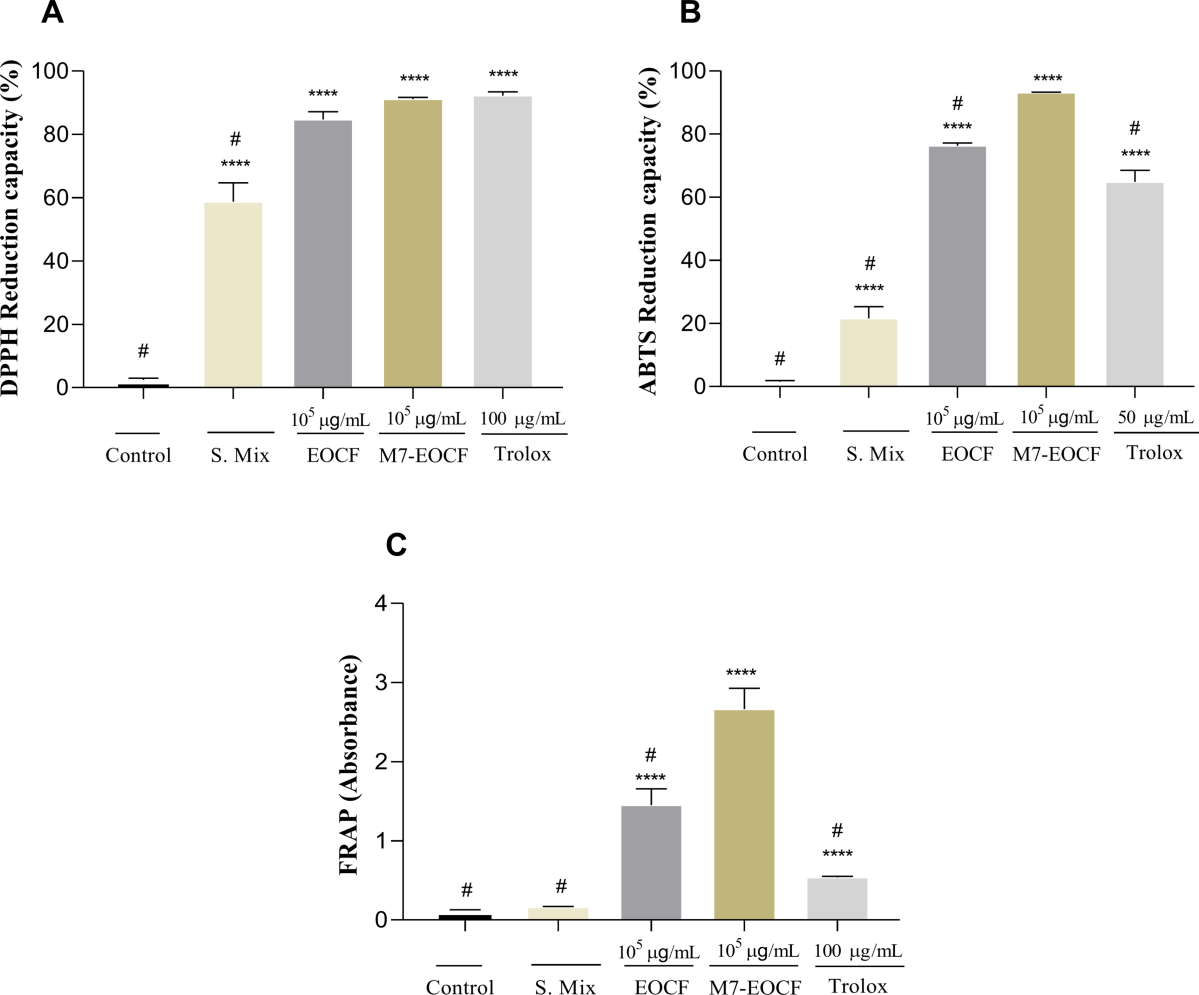
In vitro antioxidant activity of essential oil of *Cymbopogon flexuosus* (EOCF), its microemulsion (M7-EOCF), and S. Mix (surfactants + water, without EOCF). The analysis was carried out using the (A) DPPH, (B) ABTS, and (C) FRAP methods. Trolox was used as the standard antioxidant. The tests were carried out in quintuplicate, and the results represent the mean ± standard deviation of the values; ***p < 0.001, ****p < 0.0001 versus system (no antioxidants); ^#^*p* < 0.0001 versus M7-EOCF – (ANOVA followed by Tukey's test).

The results of the antioxidant activities of S. Mix (i.e., surfactants + water, without EOCF), EOCF, and M7-EOCF were evaluated using the DPPH, ABTS, and FRAP methods (Figure 3, see Experimental section for details). It can be seen in Figure 3A that M7-EOCF, EOCF, and S. Mix showed higher DPPH radical scavenging activities than the control. However, S. Mix showed significantly lower antioxidant activity (58.95% ± 5.95%) than M7-EOCF (91.12% ± 0.57%) and EOCF (84.73% ± 2.41%). Regarding antioxidant activity using the ABTS method (Figure 3B), M7-EOCF, EOCF and S. Mix also showed significant differences when compared to the control (*p* < 0.0001). The antioxidant potential of M7-EOCF was 93.12% ± 0.20%, that of EOCF was 76.28% ± 0.86%, and that of S. Mix was 21.60% ± 3.69%. Regarding the antioxidant activity determined through the FRAP method (Figure 3C), significant differences (*p* < 0.0001) were also found between the activities of M7-EOCF, EOCF, and S. Mix when compared to the control. It is also important to highlight the antioxidant and Fe^2+^ chelating potential of EOCF, as observed in a previous study by our research group [12].

Considering the results obtained, M7-EOCF showed the best antioxidant activity. It should also be noted that there was a statistical difference when compared to solely EOCF in the ABTS and FRAP methods, but not with DPPH, showing that the formulation potentiated the antioxidant activity of EOCF. This effect is probably due to the synergistic action between the chemical constituents present in the EO and the surfactants [32–33].

This difference between the DPPH method and the others can be explained by the different antioxidant mechanisms. DPPH is more useful in apolar surroundings and mainly involves hydrogen donation. ABTS is a method that includes electron and hydrogen donation, in addition to being useful for both nonpolar and polar surroundings, covering a wider spectrum of compounds [34]. Finally, FRAP evaluates the interaction of the compounds with a ferric complex (TPTZ-Fe^3+^), with hydrogen donation and reduction effects (TPTZ-Fe^2+^). Another point to be raised is the saturation of the system. In the DPPH test, the antioxidant activity of M7-EOCF is very close to the activity of Trolox, that is, above 90%. Therefore, there was possibly saturation at the concentration used.

A study by Alencar et al. [35], which aimed to analyze the antioxidant activity of lemongrass (*C. citratus*) EO before and after the development of spray-dried microcapsules, showed a reduction in the antioxidant activity of the formulation when compared to the oil alone. However, the authors defend the idea that the high temperature used in the process altered the compounds in the oil and reduced the antioxidant activity. In the microemulsion formulation in this study, room temperature was used, which may also explain why the antioxidant action was maintained.

EOCF, on *E. coli* and *S. Aureus* bacteria, showed antioxidant activity and control in pathogenic species resistant to oxidative stress. The authors also emphasized that EOCF acted as a potent attenuator of oxidative stress in in vivo models, as it has indirect antioxidants such as terpenes and terpenoids [36]. In another study using nanostructured systems containing lemongrass of the species *C. citratus*, improvements in the stability and high antioxidant content of the EO were demonstrated [37].

### Evaluation of cytotoxicity

**Figure 4:**
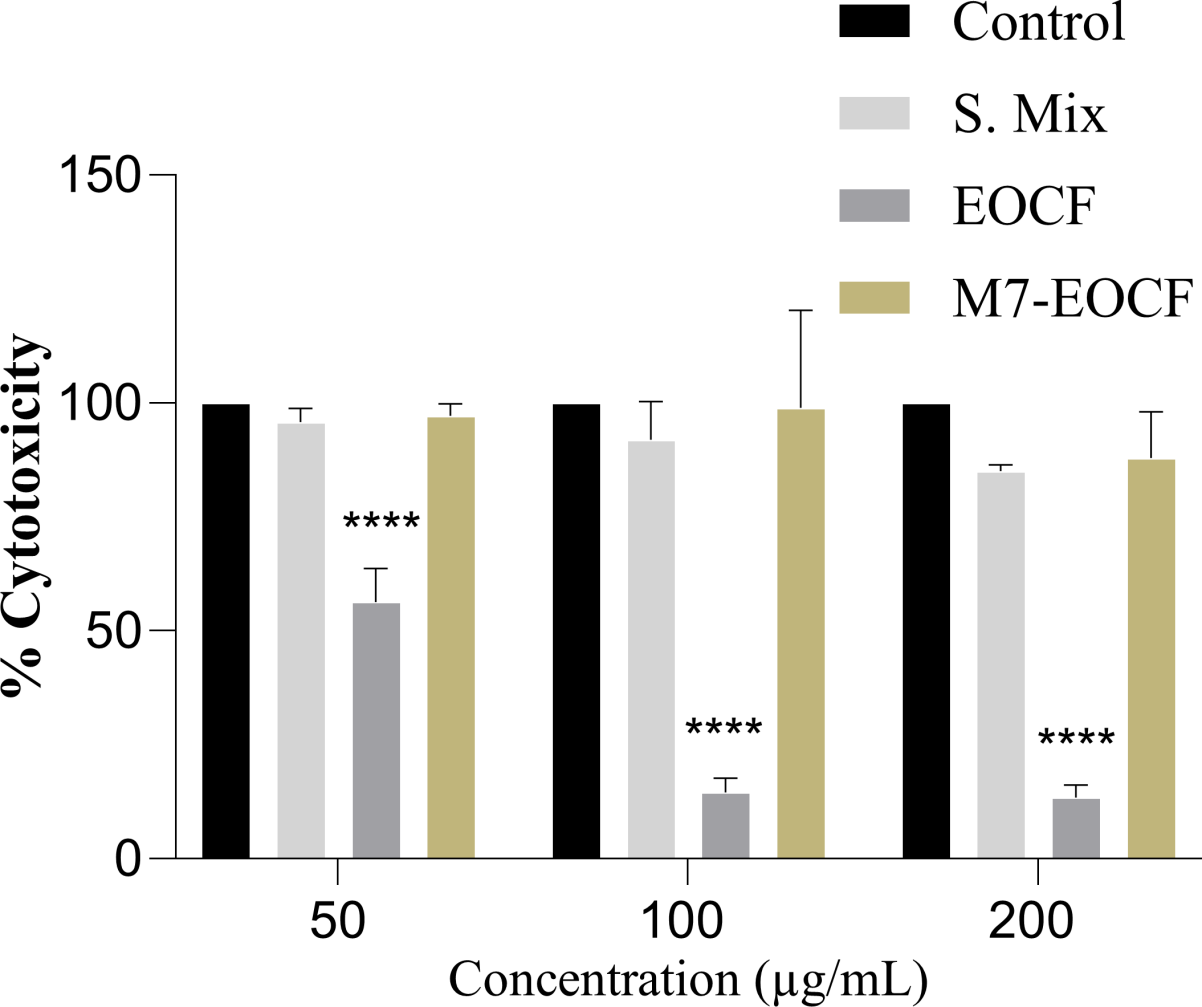
Evaluation of the cytotoxicity of essential oil of *Cymbopogon flexuosus* (EOCF), its microemulsion (M7-EOCF), and surfactant mix + water, without EOCF (S. Mix) on L929 fibroblasts using the MTT test. The results were presented as percentage of cell viability compared to the control group (culture medium). The tests were carried out in triplicate, and the results represent the mean ± standard deviation of the values; ****p < 0.0001 (ANOVA followed by Tukey's test).

The cytotoxicity of M7-EOCF, EOCF, and S. Mix was assessed through cell viability measurements on L929 fibroblasts using the MTT method [MTT = 3-(4,5-dimethylthiazol-2yl)-2,5-diphenyltetrazoline bromide]. Figure 4 shows that EOCF was considered cytotoxic at all concentrations tested (50, 100, and 200 µg/mL), with cell viability values of less than 70% when compared to the control (100% viability). The cytotoxicity of EOCF was confirmed in the study by Al-Ghanayem [38], who showed viabilities in keratinocyte cells of 51.5% and 70.5% at concentrations of 1.25 and 0.6 µL/mL of EOCF, respectively.

In contrast, S. Mix and the microemulsion (M7-EOCF) were considered non-cytotoxic at all concentrations tested, with cell viability values greater than 70%. In the study of Sá et al. [39], MEs containing the cytotoxic essential oil of *C. zeylanicum* also showed no cytotoxicity, even with a high concentration of surfactants present in the microemulsions.

### In vivo results

#### Anti-hyperglycemic activity

The aim of this study was to evaluate M7-EOCF supplementation and its effects on recovery, glycemic control, muscle damage, and reactive species markers in streptozotocin (STZ)-induced type-1 diabetic rats. The use of STZ in animals causes conditions similar to that of some humans with type-1 diabetes without glycemic control. STZ has been shown to significantly increase blood glucose levels in Wistar rats. STZ’s mechanism of action alters the DNA base sequences of pancreatic islet β-cells and stimulates polynuclear (ADP-ribose) synthetase, thus decreasing intracellular NAD and NADP levels. The depletion of NAD by STZ inhibits the synthesis of proinsulin, thus inducing experimental diabetes [40–41].

**Figure 5:**
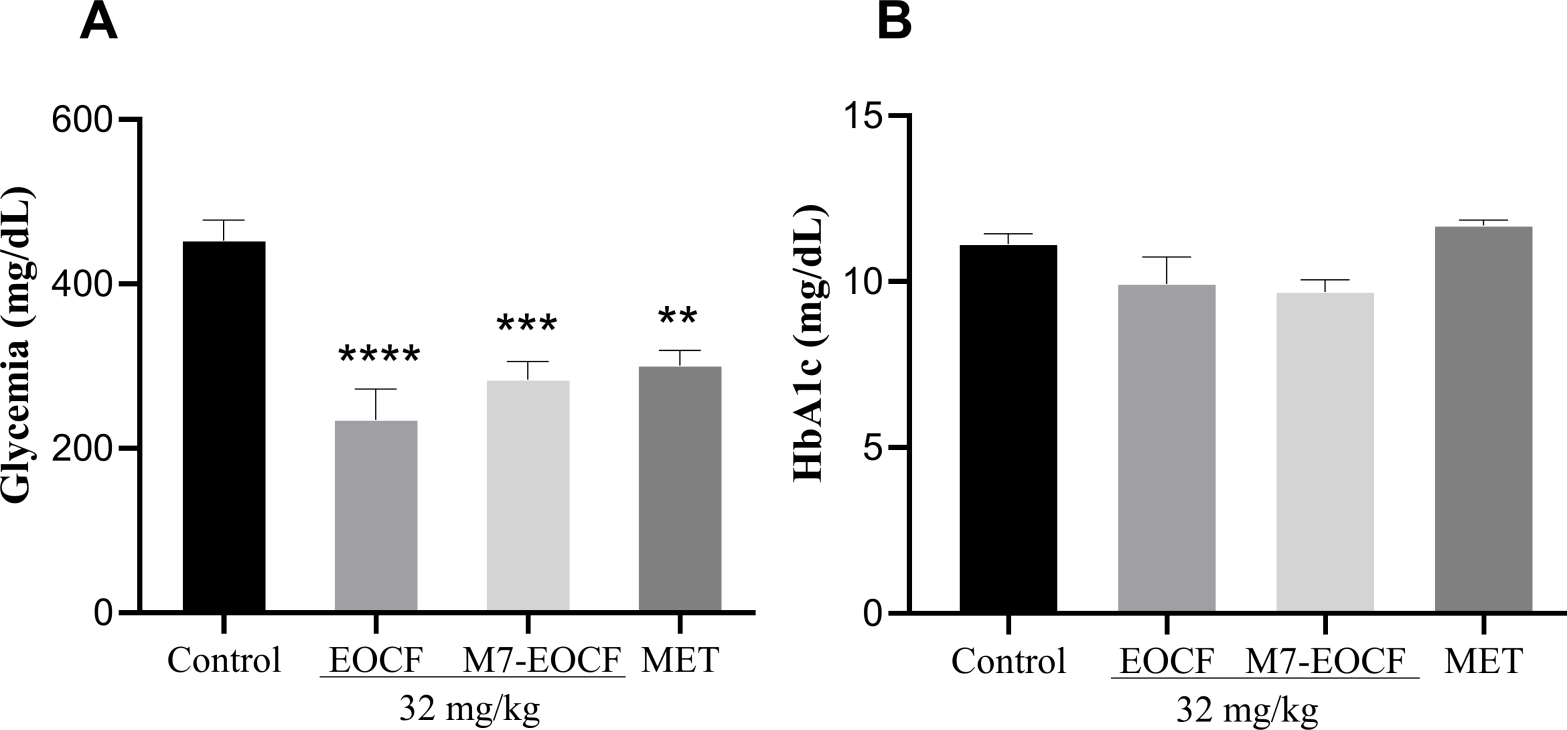
Glycemia (A) and glycated hemoglobin – HbA1c (B) levels of diabetic rats after 21 days of supplementation with EOCF (essential oil of *Cymbopogon flexuosus*) (32 mg/kg) and M7-EOCF (*Cymbopogon flexuosus* essential oil microemulsion) (32 mg/kg). Tween^®^ 80 (control) was used as a negative control. Metformin (MET) was used as a positive control. The assay was carried out in quintuplicate, and the results represent the mean ± standard deviation (SD) of the values; **p < 0.01, ***p < 0.001, ****p < 0.0001 versus control – (ANOVA followed by Tukey's post-test).

A significant increase in blood glucose levels was observed in diabetic rats in the control group (CONTROL = 452.78 mg/dL), induced by STZ, compared to supplemented animals. Treatment with the essential oil (EOCF = 234.71 mg/dL, *p* < 0.0001), the microemulsion (M7-EOCF = 283.75 mg/dL, *p* < 0.001), and metformin (MET = 300.86 mg/dL, *p* < 0.01) provided a significant reduction in glucose by, respectively, 48.16%, 37.33%, and 33.55% when compared to the control group; but there were no significant differences between EOCF, M7-EOCF, and MET. Regarding glycated hemoglobin levels, diabetic rats treated with EOCF, M7-EOCF, and MET showed no significant differences compared to diabetic animals in the control group (Figure 5B).

Corroborating the results of this research, a study carried out by Garba et al. [42], using *Cymbopogon* tea in diabetic rats, significantly reduced the induced glycemic increase. However, the data were comparable to those of the group treated with metformin, and no significant differences were observed. Sena-Junior et al. [12] evaluated the effect of citral and EOCF supplementation using different doses (32 and 64 mg/kg) in diabetic Wistar rats for 14 days. All treatments had a significant hypoglycemic effect, but with no significant differences between citral and EOCF groups.

#### Effect of EOCF on liver function

**Figure 6:**
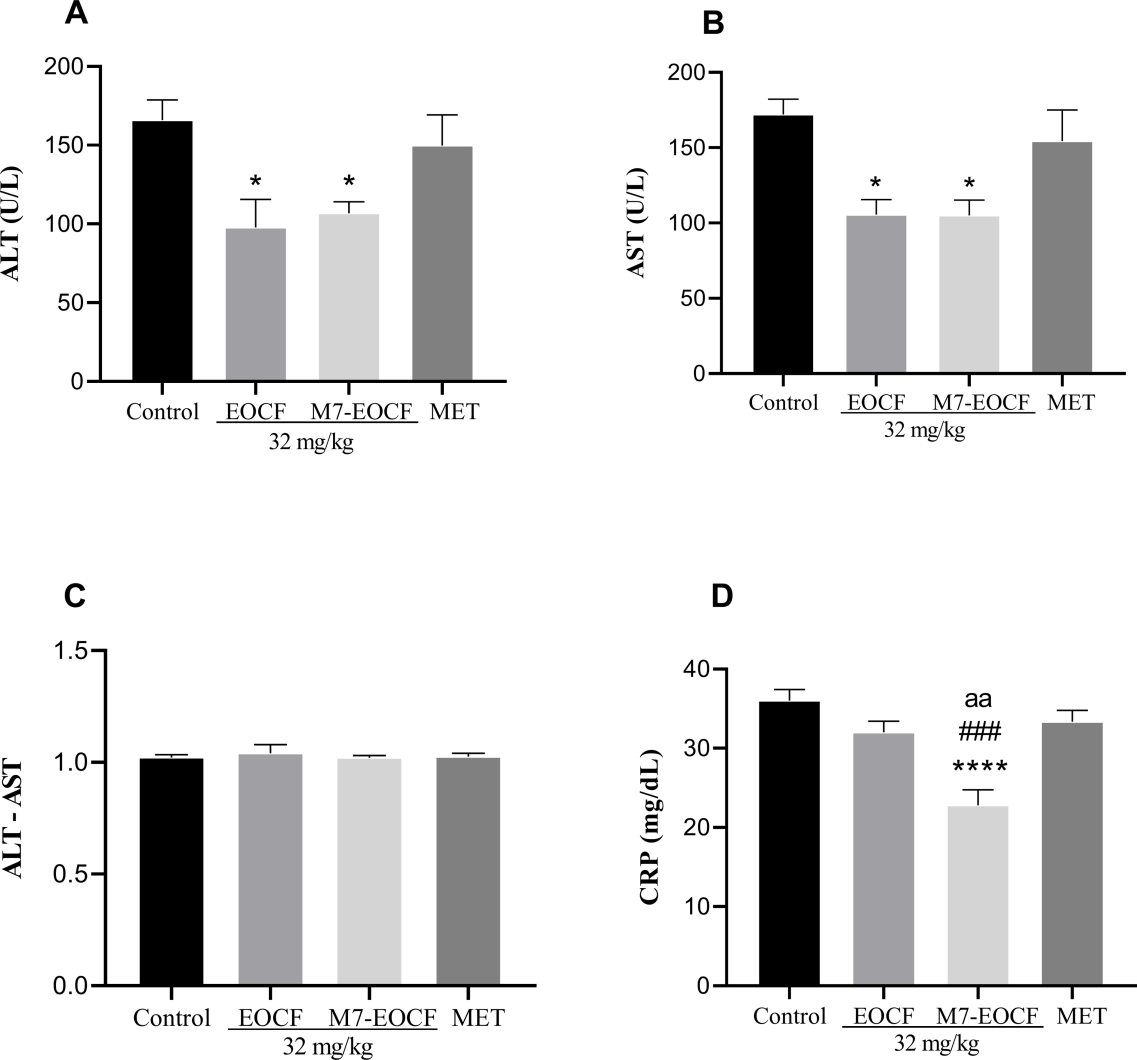
Liver levels of ALT (A), AST (B), ALT/AST ratio (C), and CRP (D) of diabetic rats after 21 days of supplementation with EOCF (*Cymbopogon flexuosus* essential oil) (32 mg/kg) and M7-EOCF (*Cymbopogon flexuosus* essential oil microemulsion). Tween^®^ 80 (CONTROL) was used as a control and metformin (MET) as a positive control. The test was carried out in quintuplicate, and the results represent the mean ± standard deviation (SD) of the values; *p < 0.05, ***p < 0.0001 versus control, ###*p* < 0.001 versus MET, ^aa^*p* < 0.01 versus EOCF (ANOVA followed by Tukey's post-test).

Exposure of rats to STZ resulted in liver dysfunction, as indicated by alanine aminotransferase (ALT) and aspartate aminotransferase (AST) activities (Figure 6). Treatments with EOCF (32 mg/kg, gavage) and M7-EOCF (32 mg/kg, gavage) significantly reduced liver dysfunction in ALT (EOCF = 97.76 U/L, *p* < 0.05 and M7-EOCF = 106.72 U/L, *p* < 0.05) and AST (EOCF = 105.61 U/L, *p* < 0.05 and M7-EOCF = 105.13 U/L, *p* < 0.05) when compared to the negative control group (CONTROL ALT = 165.98 U/L and AST = 172.10 U/L), as shown in Figure 6A,B. The positive control group (MET) did not significantly reduce ALT and AST liver dysfunction and showed no significant difference when compared to the negative control. Considering the ALT/AST ratio in all groups, the values remained below 1.5 at the end of treatment, with no significant differences (Figure 6C).

Considering the inflammation marker C-reactive protein (CRP), the M7-EOCF group (22.78 mg/dL) showed significant reductions when compared to the negative control group (36.0 mg/dL, *p* < 0.0001), the EOCF group (32.0 mg/dL, *p* < 0.01), and the positive control group (33.33 mg/dL, *p* < 0.001) (Figure 6D).

The significant reductions in ALT, AST, and CRP suggest attenuation of hepatic injury and systemic inflammation, which are commonly exacerbated in diabetic states. These effects may be attributed to the modulation of oxidative stress pathways, particularly through the scavenging of free radicals and the upregulation of endogenous antioxidant enzymes such as superoxide dismutase and catalase. The microemulsion likely enhances the bioavailability of key phytoconstituents such as citral, thereby potentiating their biological activity. To strengthen this interpretation, we included comparisons with prior studies involving lemongrass essential oil and phytocompounds in similar diabetic models, which reported concordant findings in liver enzyme normalization and inflammatory marker reduction.

In a study carried out by Falode et al. [43], the crude extract of *Cymbopogon* showed similarities to the results obtained in this work regarding the effect of EOCF on the liver functions of the animals tested. In this study, there were no significant differences between the MET-positive control group and the negative control group. Furthermore, in the same study, the induction of diabetes with STZ resulted in a significant increase in the serum and liver levels of AST, ALT, alkaline phosphatase, and gamma-glutamyl transferase as shown in the diabetic control groups compared to the normal control group.

In a study by Garba et al. [42], it was observed that liver glycogen content and serum levels of AST and ALP decreased significantly, while serum ALT, total proteins, and albumin were elevated in the diabetic control group compared to the normal control group. In addition, the group of diabetic rats treated with lemongrass tea showed a modulation in liver alterations, reverting the situation to almost normal, with a reduction in the levels of liver enzymes and markers of kidney function.

More recently, Sena-Junior et al. [12] also evaluated the effect of citral (32 mg/kg) and EOCF (32 and 64 mg/kg) supplementation for 14 days on STZ-induced diabetic rats and found that all treatments protected against ALT liver dysfunction. However, AST levels were only significantly reduced in the citral group and in the group using the highest dose of EOCF (64 mg/kg). It is likely that the present study, using the 32 mg/kg dose, found a reduction in serum levels of both AST and ALP because of the longer treatment time (21 days).

#### Effect of EOCF on metabolic lipid parameters

**Figure 7:**
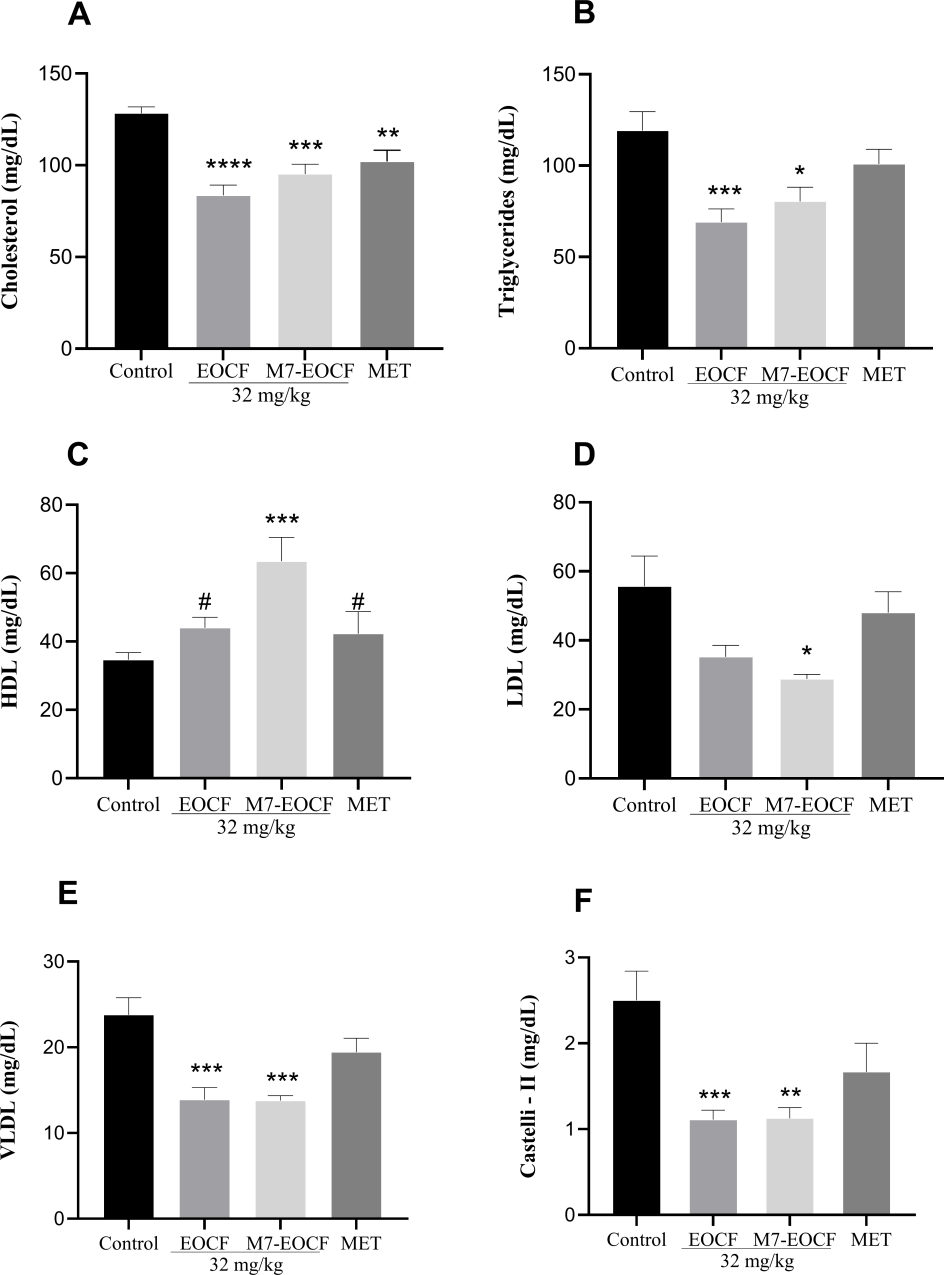
Lipid levels of total cholesterol (A), triglycerides (B), HDL cholesterol (C), LDL cholesterol (D), VLDL cholesterol (E), and Castelli-2 index (F) of diabetic rats after 21 days of treatment with EOCF (*Cymbopogon flexuosus* essential oil) (32 mg/kg) and M7-EOCF (*Cymbopogon flexuosus* essential oil microemulsion) (32 mg/kg). Tween^®^ 80 (CONTROL) was used as a negative control and metformin (MET) as a positive control. The test was carried out in quintuplicate, and the results represent the mean ± standard deviation (SD) of the values; *p < 0.05, **p < 0.01, ***p < 0.001, ****p < 0.0001 versus control, ^#^*p* < 0.05 versus M7-EOCF (ANOVA followed by Tukey's post-test).

Figure 7 shows that exposure of rats to STZ induced significant metabolic lipid disturbances. In the control group of diabetic rats, there was a considerable increase in plasma concentrations of total cholesterol (TC = 128.33 mg/dL), triglycerides (TG = 119.22 mg/dL), and low-density lipoprotein (LDL) cholesterol (155.67 mg/dL), along with a decrease in high-density lipoprotein (HDL) cholesterol levels (34.67 mg/dL) and an increase in Castelli-2 index (2.60 mg/dL). The positive control group (MET) only showed a difference in TC levels (102.11 mg/dL), with no significant change in the other metabolic parameters. The EOCF group showed significant reductions in TC (83.56 mg/dL), TG (69.00 mg/dL), very-low-density lipoprotein (VLDL) cholesterol (13.89 mg/dL), and Castelli-2 index (1.13 ± 0.35 mg/dL). The M7-EOCF group showed reductions in plasma concentrations of TC (95.22 mg/dL), TG (80.44 mg/dL), LDL cholesterol (128.86 mg/dL), VLDL cholesterol (13.78 mg/dL), and Castelli-2 index (1.14 mg/dL), as well as a significant 83.15% increase in HDL cholesterol levels (63.50 mg/dL).

Garba et al. [42] demonstrated in their study that serum levels of TC, TG, and LDL cholesterol were significantly reduced in the diabetic group treated with lemongrass tea compared to the control group. In addition, the tea further reduced HDL cholesterol, although not statistically different compared to the diabetic control. In comparison, the previous study of our group [12] demonstrated that EOCF at a dose of 32 mg/kg supplemented for 14 days significantly reduced TG and increased HDL cholesterol in diabetic rats.

In this study, oral administration of EOCF for 21 days also promoted an increase in HDL cholesterol levels. However, HDL cholesterol levels were higher for microemulsions than for the free essential oil. It is known that microemulsions promote greater bioavailability of hydrophobic molecules orally due to the nanometric size of the droplets. Additionally, nonionic surfactants such as Tween^®^ and Cremophor^®^ inhibit CYP3A metabolism or P-glycoprotein drug efflux, thus improving intestinal drug absorption. Thus, the increase in HDL cholesterol levels may be due to increased oral bioavailability [44].

Itankar et al. [45] also presented similar results to this study where the induction of DM led to a marked increase in serum levels of TG, TC, VLDL cholesterol, and LDL cholesterol, as well as to a reduction in HDL cholesterol levels. After treatment with an aqueous extract of *Cymbopogon citratus* from organically grown plants, there was a significant reduction in serum lipid levels in diabetic mice that had previously been elevated due to the induction of diabetes.

#### Effect of EOCF on kidney function

**Figure 8:**
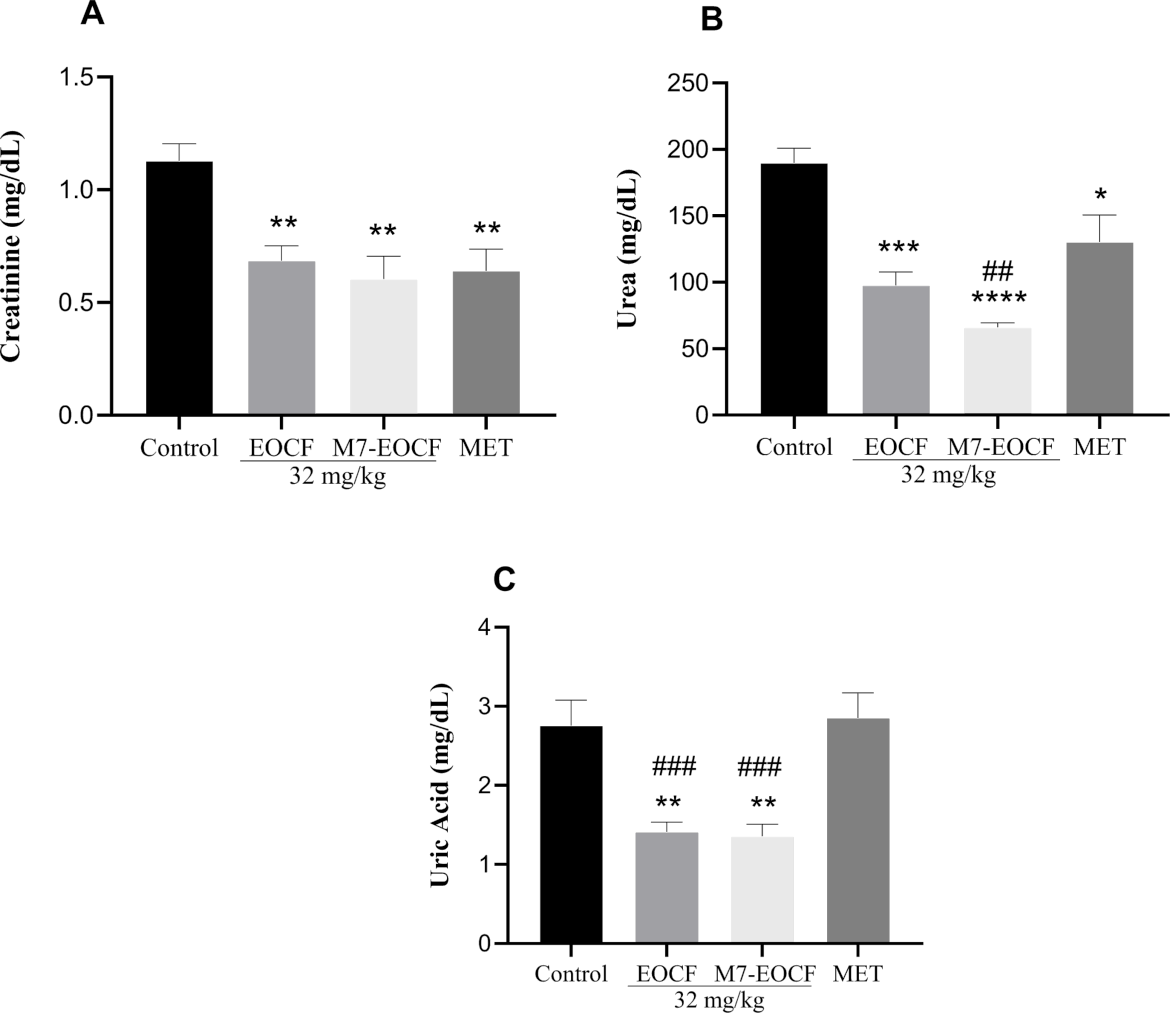
Renal markers creatinine (A), urea (B), and uric acid (C) of diabetic rats after 21 days of treatment with EOCF (*Cymbopogon flexuosus* essential oil) (32 mg/kg) and M7-EOCF (*Cymbopogon flexuosus* essential oil microemulsion) (32 mg/kg). Tween^®^ 80 (CONTROL) was used as a negative control and metformin (MET) as a positive control. The test was carried out in quintuplicate, and the results represent the mean ± standard deviation (SD) of the values; *p < 0.05, **p < 0.01, ***p < 0.001, ****p < 0.0001 versus control (ANOVA followed by Tukey's post-test); ^##^*p* < 0.01, ^###^*p* < 0.001 versus EOCF (ANOVA followed by Tukey's post-test).

Exposure of rats to STZ resulted in kidney disorders, as indicated by increased creatinine, urea, and uric acid levels (Figure 8). Treatment with EOCF (32 mg/kg, gavage) and M7-EOCF significantly protected against changes in renal markers. Treatment with M7-EOCF (Figure 8B) showed significant differences in the urea level (M7-EOCF = 64.17 mg/dL), reducing it by 66.61% when compared to the negative control group (192.20 mg/dL, *p* < 0.0001) and by 46.07% when compared to the positive control group (MET = 119.00 mg/dL, *p* < 0.01). Regarding uric acid, treatment with EOCF (1.41 mg/dL) and M7-EOCF (1.36 mg/dL) significantly reduced the values when compared to the negative control group (2.76 mg/dL) and the positive control group (2.85 mg/dL) (Figure 8C).

Regarding renal markers, Dobhal et al. [46] showed in their study an increase in uric acid levels in rats after inducing diabetes and a significant reduction in these levels in the groups treated with aqueous extract of lemongrass (100 and 200 mg/kg), ethanolic extract of lemongrass (125 and 250 mg/kg), and the essential oil of lemongrass (150 and 300 mg). Almdal and Vilstrup [47] and Mansour et al. [48] showed that induced diabetic hyperglycemia raised plasma levels of the kidney markers urea and creatinine, but there was a decrease in these markers after administration of *Cymbopogon proximus* suspension, made from ground seeds of the plant and suspended in double-distilled water. Sena-Junior et al. [12] found a reduction in uric acid levels in diabetic rats after 14 days of daily supplementation with EOCF (64 mg/kg), but the difference was not significant.

The observed decreases in urea and uric acid levels in this study indicate a nephroprotective effect. This may be due to a combination of improved glycemic control and antioxidant activity. Chronic hyperglycemia is known to induce oxidative damage in renal tissues via the formation of advanced glycation end products and increased reactive oxygen species (ROS) generation. By mitigating hyperglycemia, as evidenced by reductions in fasting glucose, both EOCF and M7-EOCF may indirectly reduce renal stress. Simultaneously, the enhanced antioxidant activity of the microemulsion, especially regarding ABTS and FRAP, suggests a direct protective role against oxidative damage. While our data suggest a dual mechanism, future studies using molecular markers of oxidative stress in renal tissue would further clarify the primary pathway involved.

### Histological assessment

**Figure 9:**
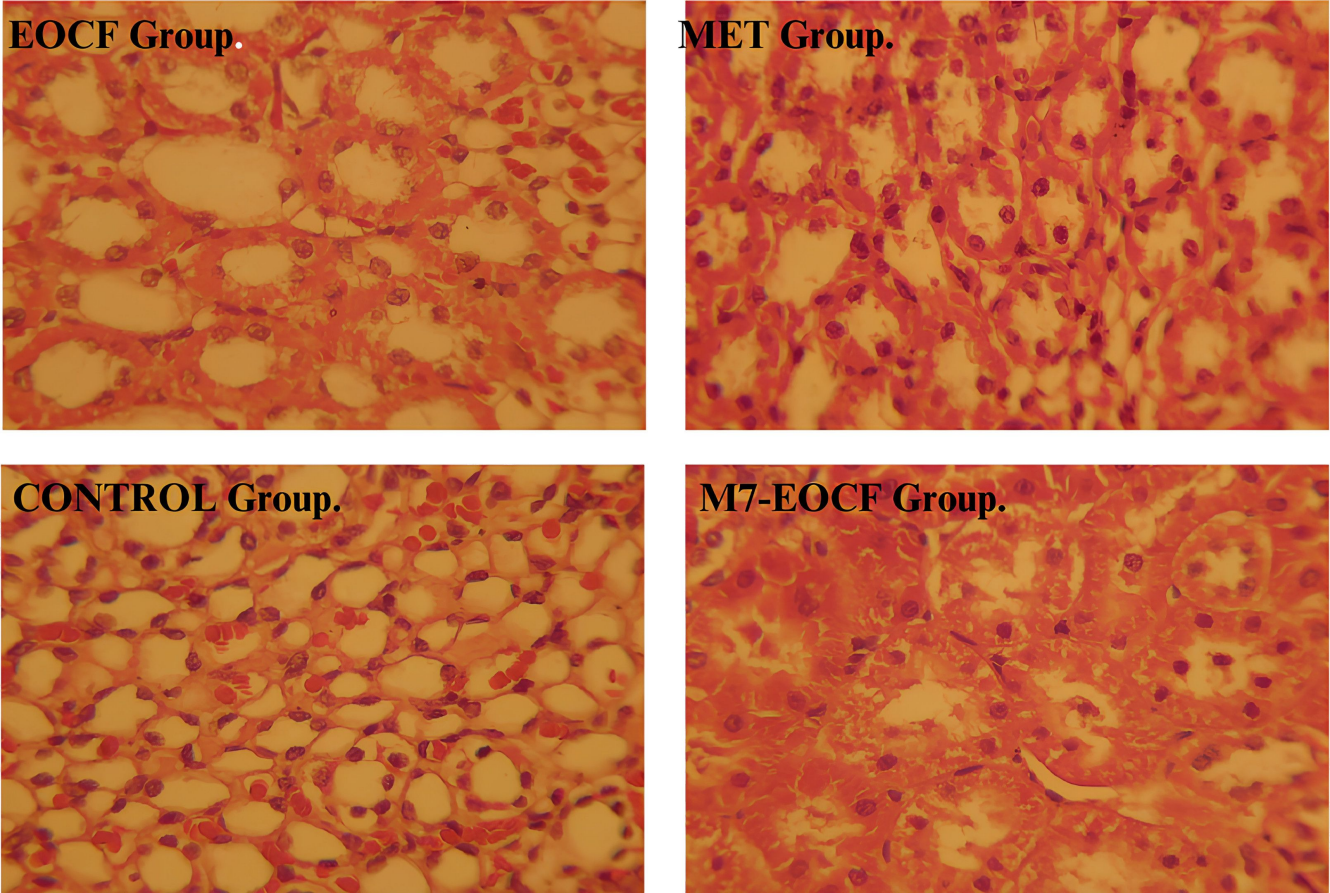
Histological sections of the liver from the experimental groups. The EOCF group shows extensive vacuolization and tissue disorganization. The MET group shows characteristics of necrosis and inflammation with signs of regeneration. The CONTROL group shows moderate damage, with less inflammation and necrosis. The M7-EOCF group is characterized by tissue preservation, less cell damage and regeneration.

Histopathological examination revealed that the EOCF group of mice exhibited extensive lesions in the liver and pancreas, with significant structural and functional impairment of these organs. Analysis of the liver sections, illustrated in Figure 9, showed morphological alterations suggestive of an advanced pathological process. In the liver, coagulative necrosis was identified in large areas of the parenchyma, characterized by the initial preservation of cell contours, but with intensely eosinophilic cytoplasm and loss of nuclear integrity. Structural disorganization was evident, with hepatocytes (H) arranged irregularly, many of them showing vacuolized cytoplasm, indicating cell degeneration, as well as pyknotic nuclei, reflecting large-scale cell death.

**Figure 10:**
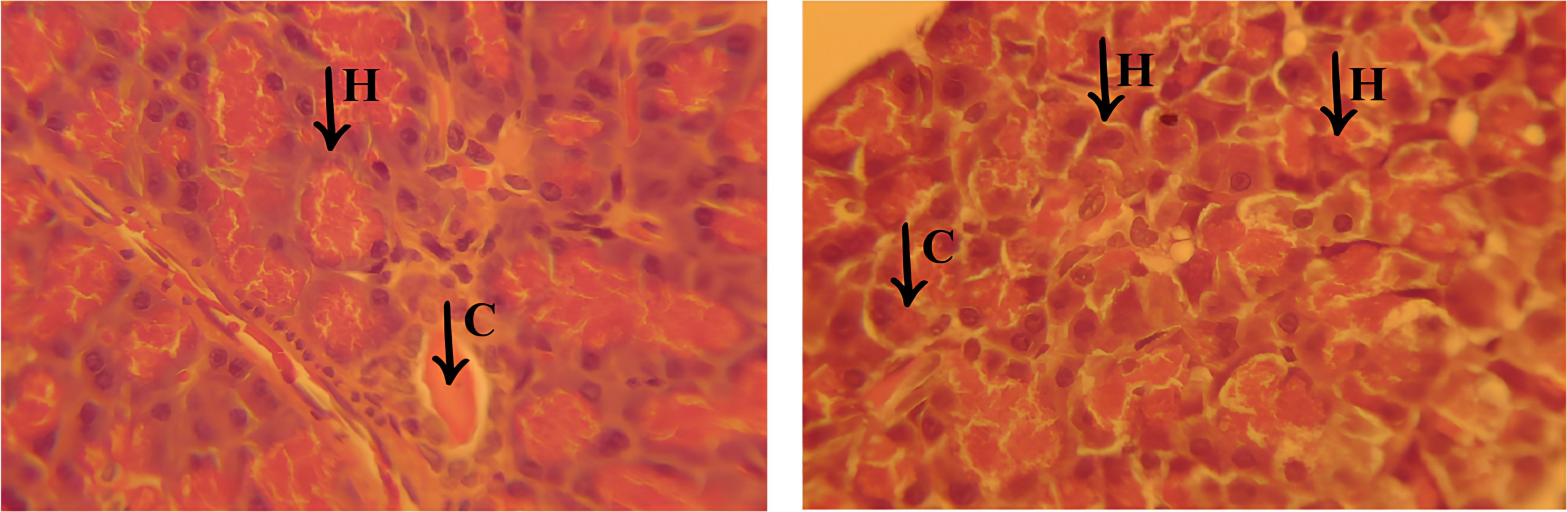
Histological sections of liver from the EOCF group. The images show areas of coagulative necrosis, characterized by disorganized hepatocytes (H), with vacuolized cytoplasm and degenerated or absent nuclei, indicating severe tissue damage. In addition, chronic inflammatory infiltrates (C) are observed, marked by cellular accumulations inside cavities, suggesting a persistent inflammatory response.

The presence of a chronic inflammatory infiltrate was a striking finding, characterized by the accumulation of mononuclear cells, mainly lymphocytes and macrophages, around the portal spaces and areas of necrosis. In addition, the formation of cavities filled with new inflammatory cells was observed (C), suggesting a persistent and progressive inflammatory process (Figure 10). This infiltration may indicate an attempt by the body to repair the damaged tissue, although in severe cases such as the one observed, liver regeneration may be insufficient to restore the organ’s homeostasis.

Another relevant aspect was the presence of vacuolar degeneration of hepatocytes, characterized by the accumulation of cytoplasmic vesicles, indicating dysfunction in metabolization and impaired cell metabolism. This finding, associated with necrosis and inflammation, suggests an exacerbated cellular response to an aggressive agent, leading to structural collapse of the liver tissue. These histopathological changes indicate that the liver has undergone a severe degenerative process, compromising its function. The combination of extensive necrosis, persistent inflammation, and tissue disruption reinforces the serious nature of the injury, as evidenced by detailed histological analysis.

**Figure 11:**
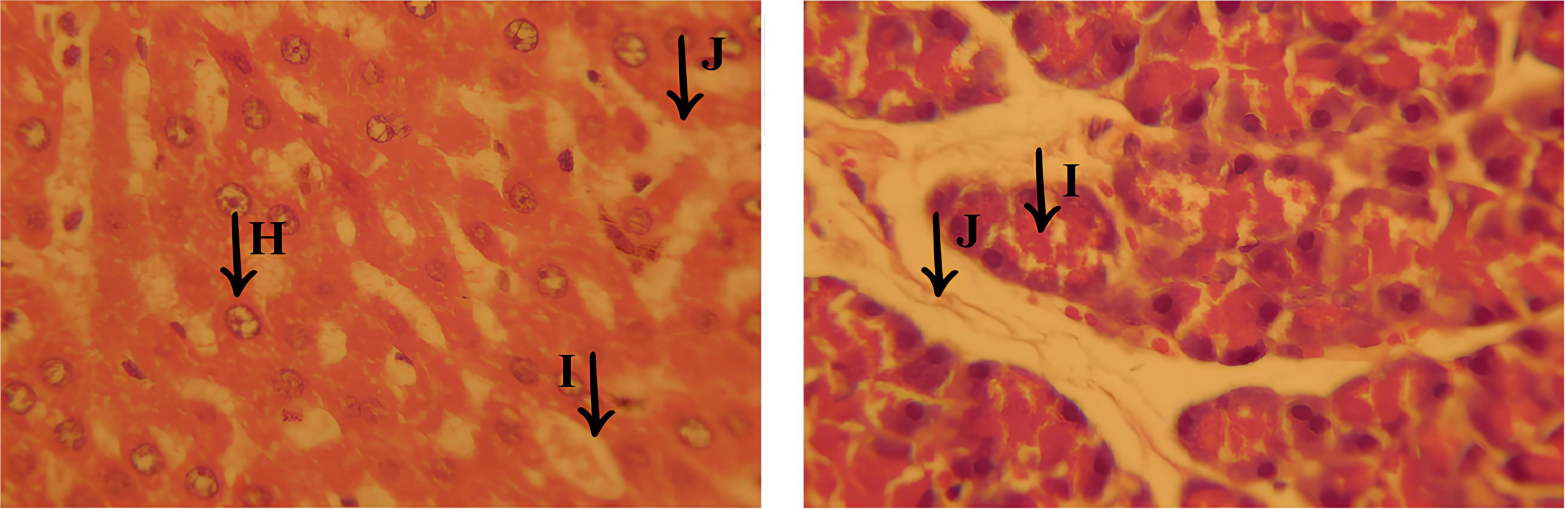
Significant lesions in the pancreas, highlighting interstitial edema (I). Interstitial edema contributes to cell spacing and cell disorganization, indicating a diffuse degenerative process. Presence of active inflammation (J) and destruction of pancreatic cells, suggesting local necrosis, with worsening interstitial edema and tissue disorganization.

Histopathological analysis also revealed significant lesions in the pancreas, as illustrated in Figure 11, with emphasis on the regions indicated by (J) and (I). One of the most obvious findings was interstitial edema, characterized by excessive accumulation of fluid in the extracellular space, which resulted in increased spacing between cells and reinforced the presence of an active inflammatory process, as well as indicating a diffuse degenerative condition (J). The cellular disorganization associated with interstitial edema was particularly pronounced in region (I), suggesting that pancreatic tissue damage was severe and progressive. The structural alteration observed, marked by the separation of cells and loss of cohesion between them, compromised the integrity of the pancreatic parenchyma, which may indicate dysfunction in enzyme production and secretion, as well as an exacerbated inflammatory response.

In addition, cell destruction associated with necrosis and local inflammation was seen in region (J). The loss of pancreatic cell integrity may have contributed to worsening interstitial edema and intensified tissue disorganization, favoring a cycle of progressive damage. The presence of these histological findings suggests an extensive degenerative process throughout the pancreatic tissue. However, the possibility should be considered that the pancreatic lesions observed in all groups were induced by streptozotocin, a cytotoxic agent selective for the β-cells of the pancreatic islets. This factor may have exacerbated the histopathological picture, intensifying cell degeneration and the inflammatory response.

**Figure 12:**
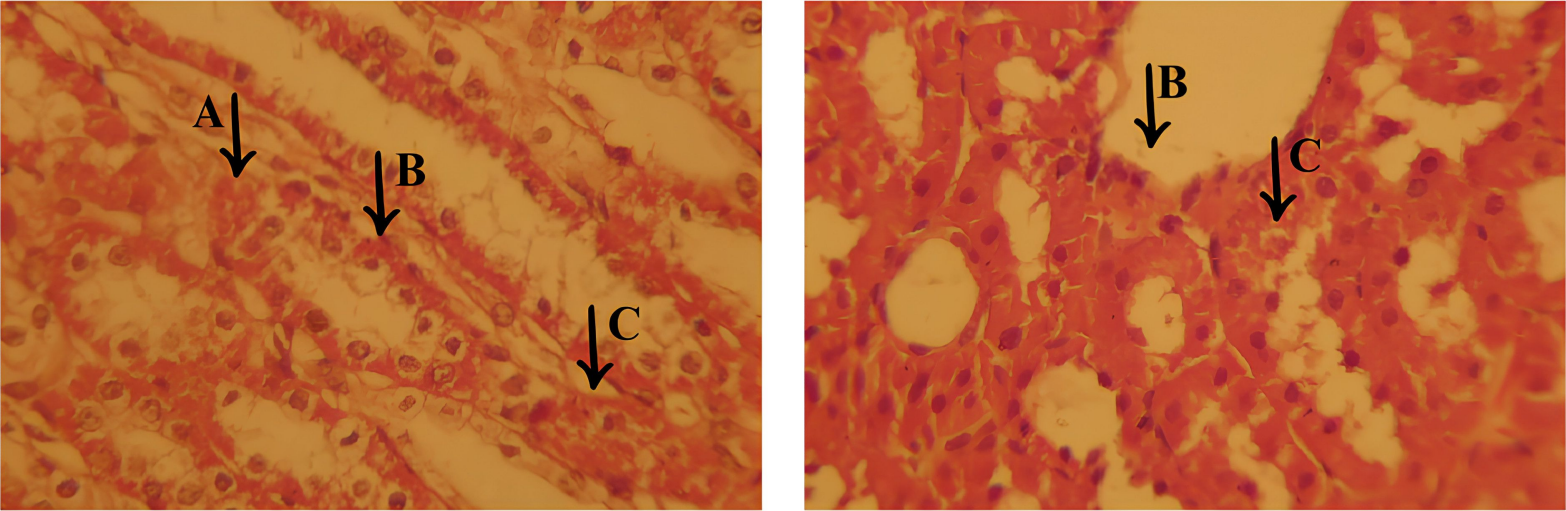
Histological section of the liver from the MET group, showing significant morphological changes. In (A), there is cellular vacuolization, indicating the presence of degenerative and apoptotic processes. In (B), there is the presence of cellular edema. In (C), there are areas of chronic inflammatory infiltrate, with cavities filled with inflammatory cells.

Histopathological analysis of the MET group (Figure 12) revealed significant cell damage in the liver, including necrosis and inflammation, but with signs of regeneration in certain areas, suggesting a reparative response to the applied treatment. Despite the considerable degree of damage, this group showed signs of attempted tissue restoration and was classified as the second most severe group in terms of the lesions observed. Important morphological changes were identified in the liver tissue. Cell vacuolization (A) was one of the most obvious findings, characterized by the accumulation of cytoplasmic vesicles, a phenomenon often associated with degenerative and apoptotic processes. This alteration reflects an impairment of cellular homeostasis and represents an important marker of liver damage in various pathological conditions.

In addition, cell edema (B) was found, characterized by swelling of the hepatocytes due to excessive accumulation of intracellular fluid. This alteration may be associated with dysfunctions in the permeability of the plasma membrane, leading to an osmotic imbalance, which favors fluid retention and compromises cell viability. Another relevant finding was the presence of areas with an active inflammatory response. Regions with inflammatory infiltrate (C) reinforce the persistence of the inflammatory process, and the body attempts to contain tissue damage. These histological findings indicate that, despite the significant damage observed in the MET group, there are signs of tissue regeneration, showing a dynamic balance between inflammatory aggression and the liver’s reparative response.

**Figure 13:**
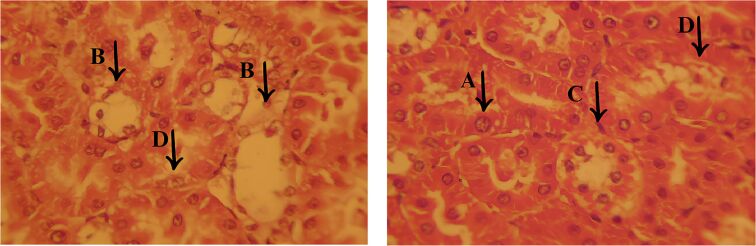
Histological section of the liver from the CONTROL group, showing moderate damage. In (A), cell vacuolization is observed, indicating moderate to mild damage. In (B), there is cellular edema with angiogenesis and mild inflammation. In (C), there is a chronic inflammatory infiltrate, with cavities filled with inflammatory cells. The areas adjacent to the infiltrate show signs of cellular degeneration (D), but to a lesser degree.

Histopathological analysis of the CONTROL group (Figure 13) revealed less inflammation and vascular congestion compared to the MET group, as well as moderate interstitial edema. Liver damage was less severe, with a lower incidence of necrosis and cell degeneration, indicating a reduced level of tissue impairment compared to the EOCF and MET groups. Histological examination showed moderate damage to the liver tissue, characterized by cellular vacuolization (A), an indication of cellular stress and sublethal damage, without significant progression to extensive necrosis. In addition, cellular edema was observed (B), with a slight increase in intracellular volume and the presence of prominent capillary vessels, suggesting a slight disturbance in tissue homeostasis.

Another relevant finding was the presence of a chronic inflammatory infiltrate (C), characterized by cavities containing inflammatory cells, although to a lesser extent when compared to the other experimental groups. The areas surrounding the infiltrate showed signs of cell degeneration (D), but to a limited extent, suggesting a controlled inflammatory response and less aggression to the liver tissue. These findings indicate that, despite the presence of moderate lesions, the control group showed less histological impairment compared to the other groups, with less inflammatory and degenerative impact, reflecting better preservation of the liver architecture.

**Figure 14:**
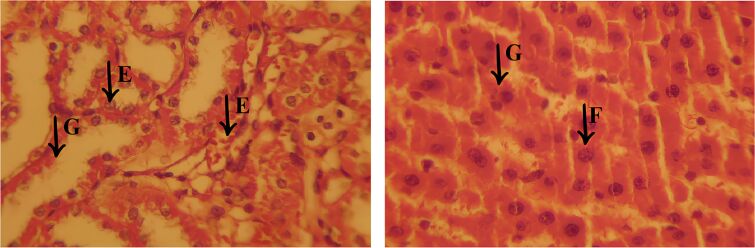
Histological section of the liver from the M7-EOCF group, showing relevant features of tissue preservation and cell regeneration. In (E), there is an attempt at cell regeneration, with proliferative and organized cells. In (F), there is vascular congestion and sinusoidal dilation, possibly due to inflammation or local injury. In (G), there are areas with partially preserved tissue architecture.

The group treated with *Cymbopogon flexuosus* essential oil microemulsion (M7-EOCF) showed the least signs of liver damage of all the groups, with minimal or no histological alterations (Figure 14). The results suggest a significant potential for tissue preservation, as evidenced by the absence of severe necrosis and inflammation.

Histological analysis revealed relevant characteristics: In region (E), there was an attempt at cell regeneration, with proliferating and organized cells. Region (F) showed vascular congestion and sinusoidal dilation, possibly secondary to inflammation or local damage. In region (G), areas with partially preserved tissue architecture were identified, where the cells had small, condensed nuclei, indicating an attempt at regeneration, albeit with a loss of cellular detail. These findings reflect a favorable tissue response in the M7-EOCF group, suggesting an effective regenerative process and significant preservation of liver integrity.

Histological data indicate that treatment with M7-EOCF provided significant protection against liver damage and inflammation induced by DM. This microemulsion appears to be a promising therapeutic approach for mitigating liver complications associated with inflammatory and oxidative conditions. The literature associates DM with oxidative damage to tissues, especially the liver, due to an increase in ROS, which cause chronic inflammation. The liver, which is essential for glucose and lipid metabolism, is particularly vulnerable to the metabolic stress generated by prolonged hyperglycemia and insulin resistance, resulting in inflammation, hepatic steatosis, and fibrosis.

Based on the discussed findings, it was possible to establish a classification of the severity of the lesions observed in the different groups. The EOCF group had the most severe injuries, characterized by extensive necrosis and severe inflammation, with no signs of regeneration. The MET group showed significant lesions, but with some degree of regeneration, indicating potential for recovery. The control group showed moderate lesions, with less pronounced inflammation and necrosis compared to the previous groups. Finally, the M7-EOCF group showed the smallest lesions, with few alterations, suggesting a state of normality. This classification provides a clearer understanding of the severity of the lesions in each group.

The essential oil of *Cymbopogon flexuosus*, especially its main component, citral, has anti-inflammatory and antioxidant properties, acting to neutralize ROS and thus reduce oxidative stress and liver inflammation. The microemulsion, as a nanoformulation, improves the bioavailability of the bioactive compounds, enhancing their therapeutic efficacy and facilitating the absorption and distribution of the essential oil.

The histological results corroborate these findings, showing a significant reduction in liver damage in the group treated with the microemulsion compared to the positive and negative control groups, as well as the treatment with essential oil, which showed more significant damage. This attenuating action of ROS-mediated inflammation suggests a reduction in the risk of liver complications associated with diabetes. In addition, biochemical analysis of the rats’ blood samples showed an improvement in liver and kidney parameters after treatment with the microemulsion. The cytotoxicity data also corroborate these observations, indicating that EOCF is cytotoxic, while ME-EOCF showed no cytotoxicity.

In summary, the microemulsion containing *Cymbopogon flexuosus* essential oil has emerged as a promising innovative therapy for the treatment of liver damage resulting from diabetes mellitus. However, more studies, especially human clinical trials, are needed to validate its long-term efficacy and better understand the molecular mechanisms involved.

## Conclusion

The selected microemulsion containing the essential oil of *Cymbopogon flexuosus* (M7-EOCF) was obtained using Eumulgin^®^ CO40 and Tween^®^ 80 as the surfactant phase; it presented itself as a transparent, liquid, and isotropic system, with nanometric droplet size, good stability, low polydispersity index and pH suitable for oral administration. It was also observed that M7-EOCF exhibited high antioxidant activity, showing that the formulation acted by potentiating the bioactive activity of EOCF. Finally, the in vivo results showed that EOCF, M7-EOCF, and metformin reduced blood glucose, but without significant differences between them. EOCF and M7-EOCF improved liver parameters, unlike metformin. M7-EOCF also had a significant protective effect against liver damage and inflammation induced by DM. In terms of lipid profile, EOCF and M7-EOCF also improved the parameters evaluated, reducing total cholesterol, triglycerides, and the Castelli-2 index. However, M7-EOCF also reduced LDL cholesterol and increased HDL cholesterol by 83.15%, showing a better effect on lipid parameters compared to EOCF. Regarding kidney function, both treatments, EOCF and M7-EOCF, showed an improvement in the parameters assessed, but M7-EOCF showed a more marked improvement in the urea marker. Metformin, in contrast, was only able to reduce blood glucose, promote a slight reduction in total cholesterol and improve the renal parameters creatinine and urea.

Overall, EOCF and M7-EOCF showed significant beneficial effects on various metabolic and renal parameters in diabetic rats. However, the microemulsion was able to improve the antioxidant activity of EOCF, maintain its antidiabetic activity, and provide additional benefits, such as improved kidney function, reduced systemic inflammation, increased HDL cholesterol levels, and a hepatoprotective effect.

## Experimental

### Material

The EOCF was obtained commercially from local suppliers (Engenharia das Essências, lot: 451 A225841), produced by Yanih Cosmetics (ANVISA Notification 25351.25600/2017-36). The citral was purchased from Sigma-Aldrich (Brazil) with a purity content of 95%. DPPH, ABTS, and trolox were also purchased from Sigma-Aldrich, Brazil. Streptozotocin (Cayman Chemical, USA) was obtained from Induslab (Brazil). Eumulgin^®^ CO 40 (BASF^®^, Brazil) and Tween^®^ 80 (NEON^®^, Brazil) were purchased from local suppliers.

### Identification of the chemical constituents of EOCF

To identify the chemical components, the EOCF was examined in a Thermo Scientific gas chromatograph (GC), Bremen, Germany, model TRACE 1310 coupled to a mass spectrometer (MS) model TSQ-9000, with TriPlus RSH automatic sampler. A NA-5MS column (60 m × 0.25 mm DI, 0.25 μm film thickness) was used for compound separation, with helium as a 99.999% pure carrier gas (White Martins SA) at a flow rate of 1 mL/min and a split/splitless autoinjector. For the analysis, a solution of EOCF was prepared at a concentration of approximately 10 mg/mL using hexane as a solvent, measured in a 1.5 mL glass vial. The sample then underwent chromatographic analysis. The ramp was programmed as follows: 60 °C – 3 °C/min – 240 °C (15 min). The injection mode was split (1:30), in SCAN mode, with a total analysis time of 75 min. For MS, the conditions were as follows: injector temperature 220 °C, detector temperature 240 °C, solvent cut-off time 2 min, electron ionization (EI) mode at 70 eV with a mass-to-charge ratio (*m*/*z*) range of 40 to 350 Da. The components of the essential oil were identified using their retention indices (RI), calculated for each constituent by injecting a series of linear hydrocarbon standards (C_8_–C_20_), under the same sample conditions, and compared with tabulated values, confirming the identification by comparing the compound spectra with the reference, presented by the NIST 107, 21 and Wiley 8 libraries [39] as shown in Supporting Information File 1, Table S1.

### Obtaining microemulsions from pseudoternary phase diagrams and selecting formulations

The components used to develop the MEs were Tween^®^ 80 and Eumulgin^®^ CO40 as the surfactant phase, EOCF as the oil phase, and ultrapure water as the aqueous phase. The combination Tween 80/Eumulgin CO40 (1:1) was chosen based on a previous paper from our research group [49]. The proportions of the components were defined using the pseudoternary phase diagram [50].

First, the surfactant phase was prepared from a mixture of Tween^®^ 80 and Eumulgin^®^ CO40 in a ratio of 1:1 (w/w) by magnetic homogenization for 24 h. The oil phase was then added to the surfactant solution, still under magnetic stirring, and homogenized for 30 min. The oil phase and surfactant solutions were prepared in proportions of 1:9, 2:8, 3:7, 4:6, 5:5, 6:4, 7:3, 8:2, and 9:1. Each solution was titrated with pre-defined volumes of water [51]. After each addition of water, the system was left to stand for 5 min, and the macroscopic properties were used to classify the systems into transparent liquid systems, transparent viscous systems, and non-transparent systems and to delimit the regions in the pseudoternary phase diagram.

Formulations containing 10% EOCF were then prepared individually (Table 1), and the dispersions were evaluated macroscopically and characterized 48 h after they were obtained.

**Table 1:** Centesimal composition of formulations M1–M8, containing EOCF (essential oil *of Cymbopogon flexuosus*) and the surfactants Eumulgin^®^ CO40 and Tween^®^ 80 (1:1 ratio).

Formulation	EOCF (%)	Surfactant (%)	Water (%)

M1	10	85	10
M2	10	75	20
M3	10	65	30
M4	10	55	40
M5	10	45	50
M6	10	35	60
M7	10	25	70
M8	10	5	90

### Physicochemical characterization of the selected microemulsions

The polydispersity index and average hydrodynamic radius of the droplets were determined by dynamic light scattering using Zetasizer Nano ZS equipment (Malvern Instruments, UK). For this purpose, about 1,000 μL of the sample, undiluted and at a temperature of 25 °C, were inserted into a cuvette suitable for carrying out the measurements [8].

The optical properties of the samples were investigated using a polarized light microscope (Olympus model BX51) equipped with a digital camera (Evolution LC Color). For analysis, a drop of the sample, undiluted, was placed on a glass slide, covered with a coverslip and analyzed under polarized light at 20,000× magnification [24].

The rheological behavior of the formulations was assessed by flow tests using the Modular Compact Rheometer (Anton Paar, MRC 302). A cone-plate geometry was used (diameter 49.9 mm, angle 1°, and gap 96 μm). The shear stress (γ) was evaluated as a function of the shear rate ranging from 0.1 to 200 s^−1^. The experiment was carried out in triplicate at room temperature and at 37 °C.

The pH analyses were carried out after diluting the MEs (1:10). A digital pH meter (model Analion PM 608) containing a glass electrode and temperature sensor (Phtek PH 3B), previously calibrated with buffer solution (pH 4.0 and 7.0), was used at room temperature [52].

### Determination of antioxidant activity

The in vitro antioxidant activities of the S. Mix. samples (surfactants + water, without EOCF), EOCF (10 mg/mL), and selected microemulsions containing EOCF (10 mg/mL) were evaluated using the 2,2-diphenyl-1-picrylhydrazyl (DPPH) free radical scavenging, 2,2′-azinobis-3-ethylbenzothiazoline-6-sulfonic acid (ABTS) radical capture, and ferric reduction antioxidant power (FRAP) methods. Trolox was used as a positive control at a concentration of 50 μg/mL for ABTS and at a concentration of 100 μg/mL for the DPPH and FRAP assays.

The DPPH method was carried out using the methodology adapted from Brand-Williams and colleagues [53]. An aliquot of 50 μL of each sample was added to 150 μL of methanolic DPPH solution (0.6 mM/L), and after 30 min, the absorbance was read in a spectrophotometer at 515 nm. The DPPH radical scavenging activity was expressed as a percentage calculated from the absorbance values of the control and the sample.

For ABTS, the methodology from Brand-Williams and colleagues was also adapted [53]. Initially, the ABTS solution was activated 16 h before the experiment by mixing 1.25 mL of ABTS stock solution and 22 μL of potassium persulfate solution. Then, 30 μL of each sample was pipetted and 300 μL of ABTS solution was added. After 6 min, the ABTS radical scavenging activity was read on a spectrophotometer at a wavelength of 734 nm and calculated from the absorbance of the control and the sample.

Using the FRAP method modified by Oyaizu [54], 9 μL of each sample, 27 μL of water, and 270 μL of FRAP reagent (a mixture of ferric chloride, TPTZ (2,4,6-tris(2-pyridyl)-*s*-triazine), and acetate buffer (0.3 M; pH 3.6) were pipetted in. The plate was incubated at 37 °C for 30 min, and the absorbance was read in a spectrophotometer at 595 nm. The results were expressed in absorbance.

### Evaluation of cytotoxicity on L929 fibroblasts

Cells of the L929 strain of mouse fibroblasts were inserted into 96-well plates (EasyPath^®^, EP-51-25244) with a final volume of 200 µL. The fibroblasts (1 × 10^5^ cells/mL cultured in Dulbecco MEM medium supplemented with 10% SFB (Gibco^®^, Thermo Fisher Scientific) and 1% antibiotic solution (5,000 UI penicillin + 5 mg streptomycin/mL, Sigma-Aldrich) were kept in an oven (5% CO_2_ atmosphere at 37 °C for 24 h) [55–56].

After this period, the adherent fibroblasts were treated with the S. Mix. samples (surfactants + water, without EOCF), EOCF, and the selected microemulsion containing EOCF (M7-EOCF), at concentrations of 50, 100, and 200 µg/mL, and cultured under the previous conditions for further 24 h. At the end of this phase, the cell monolayer was washed twice with phosphate-buffered saline, and then 200 µL of MTT (0.5 mg/mL, Sigma-Aldrich) was added to each well.

The plate was incubated again under the same conditions for 3 h. After the incubation period, the MTT was aspirated, and the formazan crystals were solubilized in 200 µL of DMSO. After placing the plate in the oven for 10 min to stabilize the color, the optical density (OD) was measured at a wavelength of 570 nm in a microplate reader. The analysis was carried out in quadruplicate, and 0.1% DMSO was considered the negative control [55–56]. The results were expressed as the percentage of viability according to the formula: %viability = (OD_570_ treated wells/OD_570_ control wells) × 100.

### In vivo study in Wistar rats using a DM1 model

#### Animals

The trials in this study were approved by the UFS Animal Use Ethics Committee, under CEUA No. 8502100821. Male Wistar rats, aged three months and weighing approximately 250 to 300 g, were used in the study. These rats were obtained from the animal house of the Physiology Department of the Federal University of Sergipe (UFS), São Cristóvão, Sergipe, Brazil. The animals (*n* = 40) were housed randomly in appropriate cages under controlled temperature conditions (22 ± 3 °C) with a 12 h light/dark cycle, providing 300 lux of light. They had free access to specific rodent food (Labina^®^) and water ad libitum and were kept in suitable conditions for 30 days of the experiment, respecting the guidelines of CONCEA – National Council for the Control of Animal Experimentation (Normative Resolution No. 12, 20/09/2013) and the minimum number of animals necessary to achieve the scientific objectives.

#### Induction of diabetes mellitus

To induce DM, the animals were fasted for 12 h to improve the sensitivity and diabetogenic action of the drug, with water supplied ad libitum.

Experimental DM1 was induced as described by Wang et al. [57], Barman and Srinivasan [58], and Sena-Junior et al. [12], using the drug streptozotocin (STZ), at a dose of 40 mg/kg body weight, dissolved in 0.01 M citrate buffer, pH 4.5, injected intraperitoneally into 40 animals. STZ was administered, and all groups were offered food 30 min later to avoid hypoglycemia. Blood was collected by tail puncture to measure blood glucose using an Accu-Chek Go glucometer (Roche Diagnostics GmbH, D-68298, Mannheim, Germany) 72 and 96 h after induction. Only animals with fasting glucose levels equal to or greater than 150 mg/dL were included in the study. After confirmation of diabetes induction, the animals were randomly assigned and treatment was started.

#### Experimental groups

Treatment took place daily for 21 days. The animals received the treatment intragastrically (by gavage) and were divided into four groups, as described in Table 2 (10 animals/group), that is, negative control group (NCG): diabetic animals treated with vehicle (saline solution + 0.2% Tween^®^ 80); EOCF group: diabetic animals treated with the EOCF supplementation solution at a dose of 32 mg/kg body weight; M7-EOCF group: diabetic animals treated with the EOCF microemulsion supplementation solution at a dose of 32 mg/kg body weight; positive control group (PCG): diabetic animals treated with metformin supplementation at a dose of 150 mg/kg body weight.

**Table 2:** Distribution of the animals used in this study according to the test to be carried out. NCG – negative control group: diabetic animals treated with saline solution + 0.2% Tween^®^ 80; EOCF group: diabetic animals treated with *Cymbopogon flexuosus* essential oil; M7-EOCF group: diabetic animals treated with the EOCF Microemulsion; PCG – positive control group: diabetic animals treated with metformin.

Experimental group	Species	Number of groups	Initial number of animals/final number of animals	Total number

NCG	Wistar rats	1	10/10	10
EOCF	Wistar rats	1	10/10	10
M7-EOCF	Wistar rats	1	10/10	10
PCG	Wistar rats	1	10/10	10

Total				40

#### Supplementation

Supplementation consisted of the administration of EOCF and the selected microemulsion (M7-EOCF) at a dose of 32 mg/kg [59–60], or metformin at a dose of 150 mg/kg, and was carried out daily at the same time for 21 days.

#### Sample collection

After a period of 24 h from the last administration of treatment on day 21, the animals fasted for 12 h and were anaesthetized with an intraperitoneal injection of a mixture of ketamine (100 mg/kg) and xylazine (10 mg/kg). Blood and tissues (pancreas, spleen, liver, and kidneys) were collected, weighed, and stored for later analysis.

#### Determination of serum biochemical markers

The blood was centrifuged at 800*g* for 15 min at 4 °C, and the serum was stored at −80 °C. Serum concentrations of triglycerides (TG), total cholesterol (TC), HDL cholesterol, LDL cholesterol, VLDL cholesterol, alanine aminotransferase (ALT), aspartate aminotransferase (AST), ALT and AST ratio (ALT/AST), C-reactive protein (CRP), Castelli-2 index, fasting blood glucose (glycemia), glycated hemoglobin (HbA1c), urea, creatinine, and uric acid were determined according to the manufacturer's procedures (Labtest^®^, Lagoa Santa, Minas Gerais, Brazil).

#### Histological evaluation

Samples of pancreas, spleen, liver, and kidneys were immersed in paraffin, followed by sectioning using a microtome. The paraffin was then removed from the samples using ethanol and xylene. After washing, the samples were stained with hematoxylin and eosin. The histological sections were analyzed by microscopy and photographed (OLYMPUS BX 45, OLYMPUS).

#### Statistical analysis

All statistical analyses were conducted using Graph Pad Prism version 5.0 and presented as mean ± standard deviation. Data was first assessed for normality using the Shapiro–Wilk test and then statistically analyzed between groups using one-way analysis of variance (ANOVA) and Tukey post-hoc tests. Statistically significant differences between the samples adopted were considered when *p* < 0.05.

## Supporting Information

File 1 Additional figures and tables.

## Data Availability

The data generated and analyzed during this study is available from the corresponding author upon reasonable request.
