# Supplementary material for: A formulation containing Cymbopogon flexuosus essential oil: improvement of biochemical parameters and oxidative stress in diabetic rats
Source: Beilstein J Nanotechnol. 2025 May 7;16:617–36. doi: 10.3762/bjnano.16.48 (PMC12067095; doi:10.3762/bjnano.16.48)
Supplement: File 1 — Additional figures and tables. [file Beilstein_J_Nanotechnol-16-617-s001.pdf]

BEILSTEIN JOURNAL OF NANOTECHNOLOGY

Supporting Information

for

A formulation containing *Cymbopogon flexuosus* essential oil: improvement of biochemical parameters and oxidative stress in diabetic rats

Ailton Santos Sena-Júnior, Cleverton Nascimento Santana Andrade,
Pedro Henrique Macedo Moura, Jocsã Hémany Cândido dos Santos,
Cauãn Torres Trancoso, Eloia Emanuelly Dias Silva, Deise Maria Rego Rodrigues Silva,
Ênio Pereira Telles, Luiz André Santos Silva, Isabella Lima Dantas Teles,
Sara Fernanda Mota de Almeida, Daniel Alves de Souza, Jileno Ferreira Santos,
Felipe José Aidar Martins, Ana Mara de Oliveira e Silva, Sandra Lauton-Santos,
Guilherme Rodolfo Souza de Araujo, Cristiane Bani Correa, Rogéria De Souza Nunes,
Lysandro Pinto Borges and Ana Amélia Moreira Lira

Beilstein J. Nanotechnol. **2025,** *16,* 617–636. doi:10.3762/bjnano.16.48

Additional figures and tables

License and Terms: This is a supporting information file under the terms of the Creative Commons Attribution License (https://creativecommons.org/licenses/by/4.0). Please note that the reuse, redistribution and reproduction in particular requires that the author(s) and source are credited and that individual graphics may be subject to special legal provisions.
The license is subject to the *Beilstein Journal of Nanotechnology* terms and conditions: (https://www.beilstein-journals.org/bjnano/terms)

Table S1: Chemical constituents of lemongrass essential oil. Lit. IR: literary retention index, Exp. IR: experimental retention index [1].

Peak	Retention time (min)	Compound	Exp. IR	Lit. IR	Relative area (%)
**1**	10.61	tricyclone	928	926	0.07
**2**	10.98	α-pinene	937	939	0.11
**3**	11.69	canphene	956	954	0.64
**4**	16.67	nonanone	1074	1090	0.52
**5**	17.96	linalool	1103	1096	0.65
**6**	20.50	isogeraniol	1158	1229	0.30
**7**	20.88	isoneral	1166	1164	0.38
**8**	21.76	isocitral	1185	1180	0.74
**9**	24.66	neral	1248	1238	19.42
**10**	24.99	geraniol	1256	1252	12.58
**11**	26.02	α-citral (3,7-dimethyl-2,6-octadienal)	1278	1318	53.21
**12**	30.63	geranyl acetate	1381	1381	4.90
**13**	32.82	caryophyllene	1432	1419	1.35
**TOTAL**					**94.87**

Table S2: Composition of formulation for the development of pseudoternary phase diagrams (PTPD).

PTPD	Surfactant phase	Oily phase	Transparent liquid system
**1**	Cremophor®/Span™ 20 (1:1)	linseed oil + lemongrass essential oil	no
**2**	Cremophor®/Span™ 20 (2:1)	linseed oil + lemongrass essential oil	no
**3**	Cremophor®/propylene glycol (1:1)	linseed oil + lemongrass essential oil	no
**4**	Tween® 80/Span™ 20 (1:1)	linseed oil + lemongrass essential oil	no
**5**	Tween® 80	linseed oil + lemongrass essential oil	no
**6**	Tween® 20	linseed oil + lemongrass essential oil	no
**7**	propylene glycol/Tween® 20 (1:1)	lemongrass essential oil	no
**8**	Cremophor ®/Span™ 20 (1:1)	lemongrass essential oil	no
**9**	Cremophor ®/Span™ 80 (1:1)	lemongrass essential oil	no
**10**	propylene glycol/Olivem® 300 (1:1)	lemongrass essential oil + isopropyl myristate	yes
**11**	Cremophor®/Tween® 80 (1:1)	lemongrass essential oil	yes

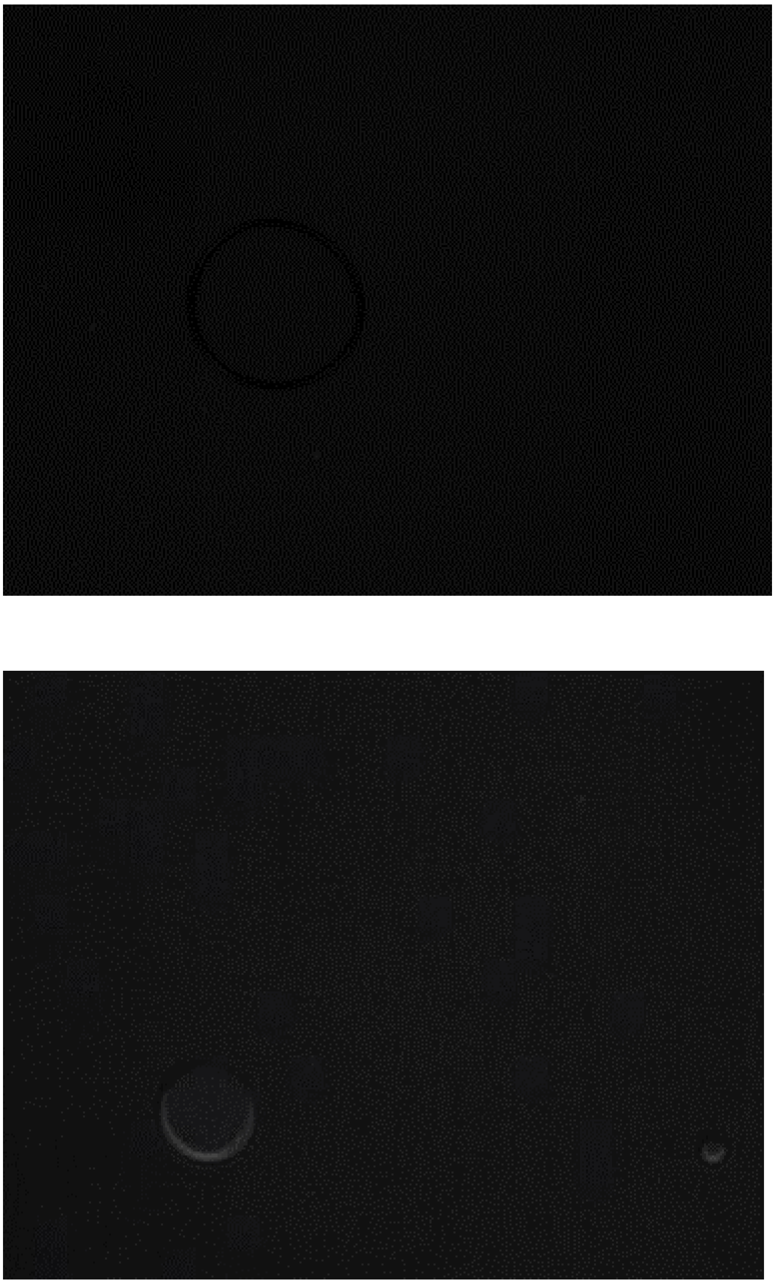

Figure S1: Images of polarized light microscopy of the M7-OECF sample representing the isotropic character of the system. Magnification of 20× (only air bubbles were seen in the sample).

References

(1) Júnior, A. S. S.; Aidar, F. J.; Silva, L. A. S.; de B. Silva, T.; de Almeida, S. F. M.; Teles, D. C. S.; de L. Junior, W.; Schimieguel, D. M.; de Souza, D. A.; Nascimento, A. C. S.; Camargo, E. A.; dos Santos, J. L.; Ana, A. M.; de S. Nunes, R.; Borges, L. P.; Lira, A. A. M. *Life (Basel, Switz.)* **2024,** *14,* 336. doi:10.3390/LIFE14030336
